# Phone-based intervention for anxiety management in primary health care users: A feasibility study*

**DOI:** 10.1590/1980-220X-REEUSP-2025-0255en

**Published:** 2026-03-02

**Authors:** Caroline Figueira Pereira, Maria Paula Bortoleti de Araújo, Divane de Vargas, Maria Luiza Prado Uelze, Geoffrey Maina

**Affiliations:** 1 Universidade de São Paulo, Escola de Enfermagem, São Paulo, SP, Brazil.; 2 University of Saskatchewan, College of Nursing, Prince Albert, Saskatchewan, Canada.

**Keywords:** Feasibility Studies, Anxiety, Primary Health Care, Mental Health, Estudos de Viabilidade, Ansiedade, Atenção Primária à Saúde, Saúde Mental

## Abstract

**Objective:**

To evaluate the feasibility of a telephone-based intervention grounded in Peplau’s Interpersonal Relations Theory for the management of anxiety among users of Primary Health Care.

**Method:**

A quasi-experimental, single-arm, before-and-after study was conducted in four Primary Health Care units in the city of São Paulo, Brazil, between January and November 2021. Feasibility was assessed across four domains: demand, acceptability, implementation, and practicality. The Interpersonal Relations Theory for the management of anxiety telephonebased intervention and its preliminary effectiveness was analyzed using a linear mixed-effects model.

**Results:**

A total of 1,327 adults were recruited, of whom 1,171 presented moderate to severe anxiety levels, identified through the State-Trait Anxiety Inventory – Six-Item Short Form. High demand (88.2%) and good acceptability were observed, with 69.8% of participants completing follow-up. A significant reduction was found in mean anxiety scores immediately after the intervention (p < 0.001).

**Conclusion:**

The telephone-based intervention demonstrated feasibility in terms of demand, acceptability, implementation, and practicality, positioning itself as a promising strategy to expand access to mental health care in Primary Health Care.

## RESUMO

**Objetivo:**

Avaliar a viabilidade de uma intervenção por telefone baseada na Teoria das Relações Interpessoais de Peplau para o controle da ansiedade entre usuários de serviços de atenção primária à saúde.

**Método:**

Um estudo quase experimental, de braço único, antes e depois, foi realizado em quatro unidades de Atenção Primária à Saúde na cidade de São Paulo, Brasil, entre janeiro e novembro de 2021. A viabilidade foi avaliada em quatro domínios: demanda, aceitabilidade, implementação e praticidade. A Teoria das Relações Interpessoais para o controle da ansiedade, intervenção por telefone e sua eficácia preliminar foram analisadas usando um modelo linear de efeitos mistos.

**Resultados:**

Um total de 1.327 adultos foram recrutados, dos quais 1.171 apresentavam níveis moderados a graves de ansiedade, identificados por meio do Inventário de Ansiedade Estado-Traço – Forma Curta de Seis Itens (the State-Trait Anxiety Inventory). Observou-se alta demanda (88,2%) e boa aceitabilidade, com 69,8% dos participantes completando o acompanhamento. Foi encontrada uma redução significativa nas pontuações médias de ansiedade imediatamente após a intervenção (p < 0,001).

**Conclusão:**

A intervenção por telefone demonstrou viabilidade em termos de demanda, aceitabilidade, implementação e praticidade, posicionando-se como uma estratégia promissora para ampliar o acesso aos cuidados de saúde mental na Atenção Primária à Saúde.

## INTRODUCTION

The World Health Organization (WHO) defines mental health as a state of well-being in which individuals can realize their abilities, cope with the normal stresses of life, work productively, and contribute to their communities. Mental disorders can profoundly affect social relationships, occupational functioning, and physical health^(1)^. According to the WHO, approximately 20% of individuals up to 25 years of age experience mental health problems, nearly twice the prevalence observed in the general population^(2)^. Alarmingly, over 85% of these individuals, particularly in low- and middle-income countries, do not receive adequate care due to barriers in accessing mental health services^(3)^. Among mental disorders, depression and anxiety are the most prevalent worldwide^(4)^. Brazil ranks fourth among countries with the highest prevalence rates of anxiety disorders^(4)^. Despite this high prevalence, only 23% of individuals diagnosed with anxiety disorders in Brazil have access to appropriate healthcare, which directly compromises their quality of life^(4)^.

Data collection for this study took place in 2021, the second year of the COVID-19 pandemic, a context in which anxiety symptoms in the Brazilian population intensified and mental health services became overloaded. Studies indicate that, during the pandemic, the prevalence of anxiety symptoms in Brazil reached levels above 40%, especially among women and young adults, groups that showed greater vulnerability to psychological distress^(5,6)^. This scenario highlighted the need for rapid, accessible, and scalable strategies for mental health care, particularly in Primary Health Care (PHC), the main gateway to the Brazilian Unified Health System (SUS).

At the same time, there was a political and institutional effort toward the digital transformation of the SUS, with the implementation and expansion of programs such as Telehealth, e-SUS, and the Digital Health Strategy for Brazil 2020–2028^(7,8)^. These initiatives sought to expand access, reduce geographical barriers, and strengthen the resoluteness of PHC, emphasizing the use of remote interventions as alternatives to face-to-face care. In this context, investigating the feasibility of interventions such as the Interpersonal Relationship in Anxiety (RIA) by telephone-based intervention becomes relevant both as an emergency response to the health crisis and as a contribution to the consolidation of innovative models of mental health care in the SUS^(9,10)^.

Telehealth has emerged as a strategic tool to strengthen Health Care Networks in Brazil, improving public health outcomes by reducing geographic barriers and facilitating the delivery of effective digital interventions^(11)^. Digital approaches for screening and managing anxiety have shown promising results in mental health care. In the current context, where the demand for mental health care has significantly increased, especially due to an increase in anxiety symptoms, it is essential to explore the potential of digital tools for managing these symptoms^(12).^

The RIA, is a structured, client-centred intervention developed by Pereira^(13)^, designed to support the management of anxiety symptoms through telephone-based intervention^(13)^. This approach is particularly suited for individuals receiving care in PHC settings, especially in low-income contexts where access to specialized mental health services is limited^(13)^. The RIA intervention is grounded in Peplau’s Theory of Interpersonal Relations, which emphasizes the therapeutic relationship as a central element in managing psychological distress. Originally adapted from the theoretical model of the Interpersonal Theory for Anxiety Management in People with Substance Use Disorders^(13,14)^, the RIA was restructured to address the broader population experiencing anxiety.

As a complex and interactive intervention, RIA aims to help individuals conceptualize their experience of anxiety and develop effective coping strategies. It comprises five essential components: (1) promoting awareness of anxiety, (2) facilitating the naming or articulation of anxious feelings, (3) identifying personal anxiety triggers, (4) recognizing existing relief behaviors, and (5) offering a menu of healthier coping alternatives. These elements are designed to enhance emotional literacy and empower individuals to respond more adaptively to anxiety-provoking situations^(13,14)^. Chart 1 provides a summary of the key elements of the RIA intervention.

**Chart 1:** Key elements of the Interpersonal Relationship in Anxiety (RIA) – São Paulo, SP, Brazil, 2025.^(13,15^

Components	Content
Component 1: Awareness of anxiety	Provide feedback on anxiety levels using the State-Trait Anxiety Inventory – Six-Item Short Form (STAI-S6) Offer psychoeducation about anxiety symptoms
Component 2: Naming anxiety	Support the participant in acknowledging and accepting their moderate or severe level of anxiety.
Component 3: Identification of anxiety triggers	Facilitate the identification of personal triggers associated with anxiety Explore determinants of anxiety
Component 4: Identification of relief behaviors	Help the participant recognize current behaviors used to relieve anxiety Promote analysis of the relationship between triggers and relief behaviors
Component 5: Menu of healthy relief behaviors	Present a menu of healthy, evidence-based strategies for anxiety management Development of skills to manage anxiety Development of self-efficacy to manage anxiety

**Figure 1:**
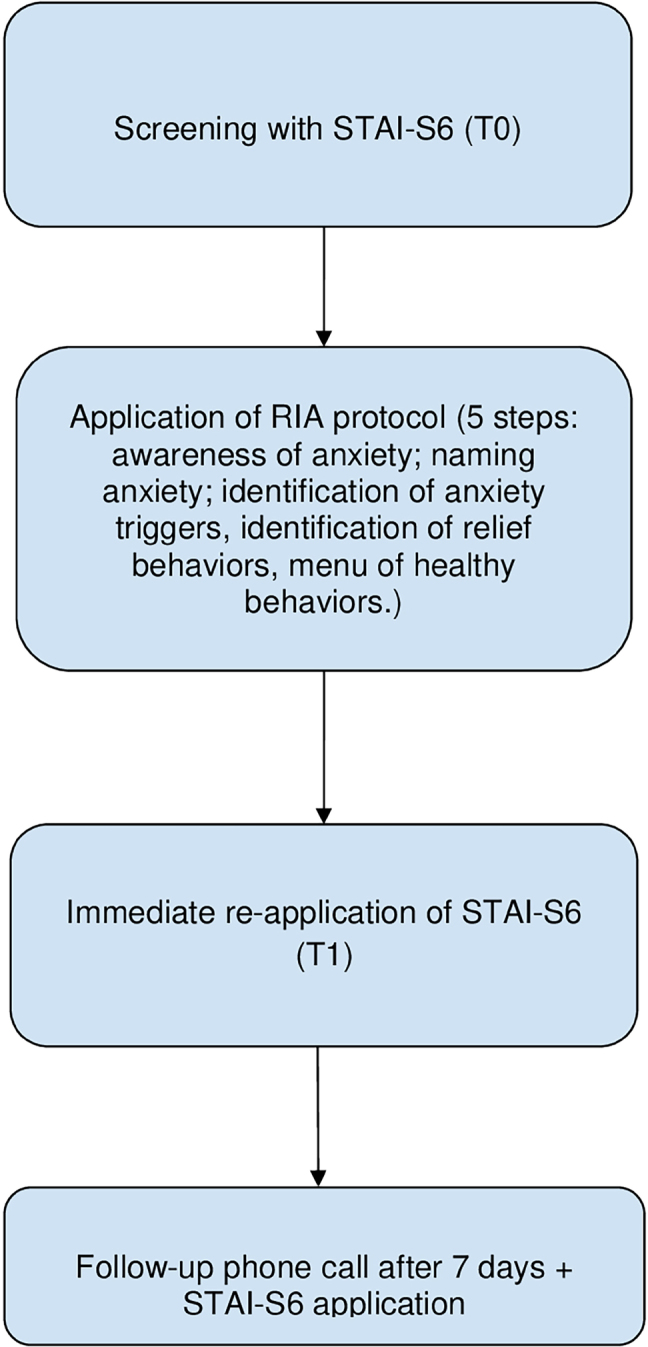
Flowchart illustrating the step-by-step application of the phone-based intervention (RIA): initial screening with STAI-S6 (T0), structured RIA protocol (five steps), immediate re-application of STAI-S6 (T1), and follow-up call with reapplication of STAI-S6 after 7 days (T2) – SP, Brazil, 2025.

During the telephone session, the State-Trait Anxiety Inventory – 6 items (STAI-S6) is initially applied to assess the participant’s current state anxiety. Subsequently, the RIA intervention is conducted in sequence, following its five structured steps. After the intervention, the STAI-S6 is reapplied to identify potential changes in anxiety levels, allowing for the immediate evaluation of the intervention’s effectiveness. The entire process, including both the scale and the intervention, takes approximately 5.7 minutes. In addition, after seven days, the RIA was reapplied along with the STAI-S6 to verify its medium-term effectiveness, as illustrated in the Figure 1 below. This brief and structured format enhances the feasibility of RIA in PHC contexts.

Despite various challenges related to health access, PHC in Brazil has employed diverse strategies to reach vulnerable populations. These include fostering close bonds between patients and healthcare teams, offering comprehensive care across all age groups; conducting community-based health promotion and prevention activities, and performing home visits^(16)^. However, the lack of appropriate tools, limited organizational support, and insufficient training among healthcare professionals often hinder the delivery of qualified mental health care. These barriers are particularly evident in the management of anxiety, where effective and empathetic communication is essential^(17)^.

In this context, the present study aimed to assess the feasibility and preliminary effectiveness of the RIA by telephone-based intervention for managing anxiety symptoms among adults, users of PHC services. The results are expected to inform the design and parameter estimation for a future full-scale randomized controlled trial (RCT).

## METHOD

### Design of Study

This is a quasi-experimental, single-arm, before-and-after study that evaluated the feasibility^(18)^ of a remote, phone-based intervention protocol for anxiety management in adults from four PHC units in São Paulo, Brazil.

A total of 1,327 individuals were recruited during appointment scheduling at the units. STAI-S6 After providing informed consent, sociodemographic data were collected, and the was administered by the research team of nurses and nursing students. The team was trained through the reading of a manual of intervention and role-play sessions with the leader of the team.

Participants with moderate to severe anxiety levels received the RIA-based intervention. Considering its medium-term effect^(19)^, a telephone-based intervention was conducted one week later to re-administer the STAI-S6 and assess the intervention’s effectiveness.

The theoretical framework for the development of the RIA was the middle range explanatory theory^(7)^. The intervention was described according to the TIDieR checklist (Template for Intervention Description and Replication), which extends the CONSORT and SPIRIT guidelines by providing clear standards for reporting interventions regarding rationale, materials, procedures, providers, and adherence strategies, thus enhancing transparency and reproducibility^(20)^. The manual of the intervention was developed to guide the interventors through a step-by-step process and is available as supplementary material.

### Scenario And Population

In Brazil, PHC adopts a dynamic, community-centered approach, characterized by in-depth knowledge of the territory, strong patient-provider relationships, comprehensive care, and proactive health promotion and disease prevention activities. Home visits are essential for closely monitoring patients’ needs. A study highlights that this structure has been crucial in emergency situations in the country^(16)^. For this reason, the study’s target population consists of adults aged 18 to 90 who are monitored by PHC units in the city of São Paulo.

The PHCs units located in the neighborhoods of Sé, Cambuci, República, and Santa Cecília—all situated in the city of São Paulo, in the state of São Paulo, Brazil—were selected for this study due to their location in low-income areas characterized by social vulnerability and complex patient care challenges.

### Inclusion and Exclusion Criteria

The inclusion criteria were: (a) being over 18 years of age, (b) having had a consultation in the PHC, (c) understanding enough Portuguese to comprehend the interviewer’s questions, (d) presenting a score equal to or greater than 11 on the STAI-S6 scale, and (e) being available for follow-up. Exclusion criteria included: (a) undergoing treatment for problems related to alcohol use and/or other mental disorders, (b) patients who show noticeable signs of mental confusion or intoxication by psychoactive substances during the phone call, (c) the hearing impaired, and (d) patients who refuse to receive the intervention and/or follow-up.

### Sample and Collection Procedures

We recruited 1327 users of PHC from January 2021 to November 2021 through the consultation and assistance schedule of the PHC units involved. A sample of 75 participants was the target, as this number is acceptable for a feasibility _study_(14,21)_._

The sample was selected by convenience, and trained interveners contacted potential participants via phone to explain the study. All data collection occurred through 20–30-minutes phone calls, during which sociodemographic, clinical, and behavioral data were collected, the STAI-S6 was administered, and the telephone-based intervention when applicable. Data was entered directly into the Research application and the Electronic Data Capture platform (REDCap), a secure web-based platform for research data management.

Collected information included gender, race, marital status, employment, education, comorbidities (diabetes, hypertension, COVID-19), and physical activity. The STAI-S6, a validated six-item scale (Cronbach’s α: 0.93 for males, 0.87 for females)^(22)^, was used to screen for anxiety, with scores ranging from 6–10 (mild), 11–15 (moderate), and 16–24 (severe).

Eligible participants received the RIA by telephone-based intervention (T0), followed immediately by a second STAI-S6 application (T1) to assess short-term impact (mean interval: 5.7 minutes). A follow-up call was conducted after seven days (T2) for reassessment and evaluation of sustained effects.

### Data Analysis and Treatment

Data was collected and managed in the REDCap and analyzed in the statistical program R version 4.0.2^(23)^. The descriptive analysis was performed through the analysis of absolute and relative frequencies. To evaluate the preliminary effectiveness of the RIA, the linear mixed effects model was used. The significance level adopted was 5%.

### Ethical Aspects

This study was approved by the São Paulo University School of Nursing Ethics Review Board (24461219.9.0000.5392/2019) and by Municipal Health Department of São Paulo (4.342.492). Furthermore, verbal informed consent was obtained from all participants, which was recorded. The verbal informed consent procedure was approved by the São Paulo University School of Nursing Ethics Review Board. Participation was voluntary, and participants were informed that they could withdraw from the study at any time. All methods used in this study followed the Ethics Review Board guidelines and regulations.

## RESULTS

### Participants

A total of 1,327 adults were screened for eligibility. Among them, 144 individuals (10.85%) declined to participate, most commonly citing a lack of interest in the study topic. The final sample comprised 1,183 participants, of whom 710 were women (60.01%), 472 were men (39.89%), and one participant (0.01%) chose not to disclose their gender.

The majority of participants were single (n = 443; 38.06%) and self-identified as white (n = 485; 41.00%). The mean age of the sample was 48.97 years. In terms of education, a substantial portion had completed high school (n = 425; 37.09%), and 411 individuals (35.31%) reported being employed at the time of the study.

### Feasibility Assessment

Robust measurements of feasibility, such as demand, acceptability, implementation, and practicality, were assessed^(21,24)^. Chart 2 informs us which components were used to assess the feasibility of the RIA intervention.

**Chart 2:** Assessment of the components of the feasibility of the RIA program – São Paulo, SP, Brazil, 2025.

Components	Indicators
Demand	Willingness to participate – (%) of participants that accepted and (%) that did not agree to participate of study Screening - (%) of participants that are ineligible according inclusion criteria
Acceptability	Follow-up rates – (%) of participants that complete the follow-up Missing rates – (%) of missing data Attrition - (%) of participants that participate of intervention across the time periods (T0, T1, e T2)
Implementation	Design - Evaluation of answers referring to a question: “Is there anything that you would change in the intervention you received via telephone?”
Practicality	Ability of participants to implement RIA strategies to manage anxiety.

The first feasibility criterion, demand, was assessed by calculating the proportion of individuals who agreed to participate following the initial screening process. A total of 1,327 adults were screened based on the study’s eligibility criteria. Among these, 144 individuals (10.85%) declined participation, with the majority (n = 46; 70.77%) citing a lack of interest in the topic. As a result, 1,183 individuals initially agreed to participate. Of these, 1,171 met the full eligibility criteria, which included consent to participate and presenting moderate to severe anxiety symptoms based on the STAI-S6 scale. This resulted in a final participation rate of 88.2%, indicating high demand for the intervention.

The component 2 (Acceptability) was assessed by follow-up rates, missed rates and attrition. When carrying out a descriptive analysis of the STAI-S6, it is possible to verify the distribution of the subjects who answered the calls. In the screening phase, we contacted 1327 (100%) people, of these 1183 (89,14%) accepted to participate in the research. Of these, 1171 (88.22%) had moderate or severe anxiety and therefore accepted the RIA intervention (T0). During the intervention, of 1171 participants who agreed to participate, 606 people (51.75%) started to answer the STAI-S6 post-intervention (T1), but only 468 (77.22%) answered the STAI-S6 post-intervention completely. Of these 468 people, 72% had moderate anxiety and 28% had severe anxiety. Finally, of the participants who completed the scales, 327 (69.87%) completed the follow-up, that is, they answered the second call and answered the STAI-S6 scale 7 days after the intervention was applied.

Implementation (component 3) was assessed by asking participants at the end of the follow-up call whether they would suggest any changes to the intervention. It can be noticed that of the 1171 participants, only 92 answered this question. Of these, 72 (78.26%) reported that they would not change the intervention received, demonstrating good acceptance of the RIA. Despite this, some suggestions such as: the intervention being face-to-face (n = 4, 4.35%) and dealing with more subjective issues (n = 3, 3.26%) were approached during telephone-based intervention. It is worth mentioning that this question was not answered by the vast majority of participants (n = 1091, 92.22%), which should be considered in the evaluation of implementation and, mainly, to improve the study.

Regarding feasibility component 4 (Practicality), the evaluation of this component included the participants’ ability to implement the anxiety management strategies suggested during the implementation of the RIA, such as physical exercise, breathing techniques, good nutrition, listening to music, crafts, and conversation to friends and family, as well as assessing whether they were useful. The results showed that, of the 92 people who answered to this question, 70.65% (n = 65) of the people reported that they were able to implement the techniques in their daily lives and that they helped to manage anxiety.

### Preliminary Effectiveness

The results suggest a positive effect of the RIA in reducing anxiety symptoms among the participants, statistically significant differences were observed between the times of application of the STAI-S6 scale, with a decrease of 0.45 points on average at T1, however, no change was observed of scores at T2 (Table 1). These results are consistent with the results presented in Table 2, which indicate that the difference between the study times was significantly greater between T0 and T1, which is not observed in the comparison with T2. However, as this is a feasibility study, a control group was not used to verify the effectiveness of RIA. These questions will be clarified in the future in a randomized clinical trial.

**Table 1:** Result of the linear mixed effects model to comparison of the STAI – S6* at baseline, T1 and T2 regarding the RIA for anxiety – São Paulo, SP, Brazil, 2025.

Time	N	Mean	P-value^†^
Baseline (T0)	117 1	12.47	
STAI-S6 post (T1)	468	11.23	<0.001
Follow-up (T2)	327	10.86	

^*^STAI-S6 = Scale Trait Anxiety Inventory – S6;

^††^ Linear Mixed Effects Model

**Table 2:** Two-by-two sequential comparison of the three measures of STAI – S6^*^ at baseline, T1 and T2 regarding the RIA for anxiety – São Paulo, SP, Brazil, 2025.

Time/Intervention	Coefficient	SE	Lower CI	Upper CI	P-value
STAI-S6^*^ baseline (T0) vs STAI-S6 post (T1)	–0.453	0.146	–0.739	–0.166	0.002
STAI-S6 post^*^ (T1) vs STAI-S6^*^ (T2)	–0.005	0.176	–0.350	0.341	0.979

## DISCUSSION

The drastic changes in daily life and the increase in stressful situations over the years have caused serious consequences for the population’s mental health, such as the rise in anxiety disorders, as documented by various authors^(5,6,7)^. In light of this scenario, it has become increasingly necessary to closely monitor the population’s mental health, given the high risk of psychological distress and the fact that many individuals may struggle to cope with these adversities^(7)^.

The findings of this study reflect this broader context, indicating a representative number of individuals with moderate to severe anxiety symptoms. A national survey revealed that 40% of adults reported frequent feelings of sadness, and when specifically analyzing anxiety symptoms, this number approaches 50%^(8)^. Furthermore, sadness and anxiety are particularly prevalent among women and young adults^(25)^, a pattern also observed in this study. These observations reinforce the urgency of developing targeted strategies to address anxiety, especially in high-reach healthcare settings such as PHC.

In this regard, the sociodemographic profile of the study participants also reveals structural and social determinants that may not only influence the onset and severity of anxiety but also affect the feasibility of implementing anxiety management strategies in daily life. In our sample, there was a predominance of women (60.01%), individuals who self-identified as white (41.00%), and single participants (37.44%). A considerable portion had completed high school (37.09%) and a slightly larger proportion reported being employed (35.31%) vis-à-vis those unemployed (29.98%).

These indicators highlight important social determinants of mental health. Evidence suggests that women are more affected by anxiety disorders due to multiple factors, such as hormonal variations and greater exposure to caregiving and domestic responsibilities^(25)^. Similarly, unemployment is associated with psychological distress, often linked to financial insecurity and the absence of a structured routine^(26)^. Educational level also plays a determining role, as it influences access to health information, comprehension of self-care recommendations, and the ability to integrate new practices into everyday life.

When analyzed in relation to the feasibility of RIA, these determinants may facilitate or hinder the adoption of suggested strategies, such as physical activity, healthy eating, or relaxation techniques. For instance, individuals with limited financial resources may face barriers to maintaining adequate nutrition or accessing spaces for exercise, while those with greater educational resources may find it easier to understand and apply breathing techniques or other coping strategies. Therefore, beyond individual motivation, the social context is a decisive factor in shaping how interventions are experienced and sustained^(27,28)^.

These findings underscore the need for public health strategies that are sensitive to sociodemographic vulnerabilities, ensuring that interventions like RIA are adaptable and inclusive, particularly within the PHC setting^(27,28)^.

PHC plays a central role as the main gateway to Brazil’s SUS. It is responsible for welcoming, monitoring, and coordinating patient care over time and is capable of addressing up to 90% of the population’s health demands^(29)^. Therefore, the implementation of systematic strategies to screen for anxiety symptoms in Basic Health Units (UBS) may promote early detection and timely interventions, preventing symptom escalation and reducing the need for referrals to specialized services^(30)^.

Strengthening the capacity of primary care services to manage common mental disorders such as anxiety can also play a decisive role in preventing the overload of specialized mental health services. Brief psychosocial interventions such as psychoeducation, cognitive behavioral therapy strategies adapted to PHC, and the use of digital technologies have demonstrated effectiveness in managing mild to moderate anxiety cases^(31)^. Evidence from Brazil reinforces the feasibility of psychosocial interventions in PHC settings, such as mindfulness and internet-based cognitive behavioral therapy^(32,33)^. Integrating these approaches into the daily work of Family Health Strategy (FHS) teams expands access to care, promotes user autonomy, and strengthens the problem-solving capacity of the primary care network. This perspective is aligned with the principles of the Psychosocial Care Network (RAPS) and the guidelines of Brazil’s public mental health policies^(34)^.

The structured anxiety management intervention (RIA) was conducted by nurses and nursing students who were specifically trained to deliver the protocol. Peplau’s theory highlights the centrality of the therapeutic relationship as a mediator in the process of coping with psychological distress, reinforcing the adequacy of this framework for mental health practice in PHC^(35)^. The training of the professionals included remote workshops, role-playing, and continuous supervision, which ensured adherence to the protocol and reinforced its feasibility. Similar strategies have proven effective in digital mental health interventions delivered in Latin America^(36,37)^. This characteristic also suggests that the intervention could be incorporated into continuing education programs for PHC nurses, contributing to the sustainability of the proposal.

For the RIA to be sustainably incorporated into PHC routines, especially within the FHS, some adaptations are necessary. These include: (1) specific training for nurses to ensure fidelity in applying the protocol; (2) consideration of time constraints and work overload faced by PHC teams; (3) integration with existing mental health screening protocols to avoid duplication and optimize workflows; and (4) the use of digital health technologies within SUS, such as PHC electronic medical records (e-SUS) and Telehealth programs (Telessaúde), which are strategic tools for scalability, continuous monitoring, and sustainability of the intervention^(38,39)^.

In addition, the detailed description of the intervention followed the recommendations of the TIDieR checklist, which provides structured guidance to ensure transparency, clarity, and reproducibility of health interventions. This methodological rigor is crucial to enable the replication of experiences such as RIA in other primary care contexts, while respecting local specificities^(20)^.

The implementation of the RIA intervention proposed in this study within PHC demonstrated initial effectiveness in reducing anxiety symptoms, in addition to showing strong potential for population-level reach. The high adherence and effectiveness of the strategies used during remote calls reinforce the feasibility of the intervention. These findings are consistent with evidence supporting the use of telephone-based intervention to identify anxiety symptoms and aggravating factors, thus contributing to improved quality of life for individuals. However, it is important to recognize that, despite the many advantages of telephone-based intervention, it does not replace in-person treatment^(36)^.

Regarding the feasibility criteria evaluated in the study, the intervention met the demand: 1,183 individuals agreed to participate, with 1,171 (88.2%) meeting eligibility criteria. This indicates that a substantial portion of the PHC-served population presented moderate to severe anxiety symptoms. According to the literature, even smaller samples (around 39 participants) are considered sufficient for feasibility studies and can inform adjustments to the clinical and sociodemographic profiles of participants for future randomized clinical trials^(40)^.

The acceptability of the RIA intervention was also notable, with 69.87% of participants completing follow-up. Although there was some loss to follow-up, the rates remained within acceptable limits for clinical trials^(41)^. The success of the implementation was evidenced by the high levels of participant satisfaction with the intervention received, and its practicality was demonstrated by how easily participants applied the techniques suggested by RIA in their daily lives. Notably, 78.26% of participants stated they would not change anything about the intervention, and 70.65% reported routinely using the techniques proposed to manage anxiety. However, it is important to highlight that 1,079 individuals did not respond to the subjective questions at the end of the consultation, which affected the full assessment of implementation and feasibility. This gap may have occurred due to participant fatigue, since the questions were inserted at the end of the consultation and followed the application of several scales, a difficulty frequently reported in feasibility studies^(41)^.

In light of these challenges, adopting a more pragmatic approach to guide complex interventions is recommended^(41)^, as well as reorganizing the timing and format of the scale applications to reduce participant fatigue and improve response rates. Additionally, ensuring data security, including integrity, accuracy, confidentiality, privacy, and professional confidentiality is essential, especially in the context of telehealth, and should be treated as a central aspect in the implementation of remote mental health care strategies^(41)^.

The objective of a feasibility study is to evaluate if the intervention works in a real scenario, what impact causal inference, making it difficult to determine whether the observed results were exclusively due to the intervention. For these reasons the preliminary effectiveness should be analyzed with caution. Future randomized controlled trials will be necessary to confirm its efficacy. In addition, participant attrition during follow-up and the low response rate to subjective questions limited the assessment of acceptability and practicality. Possible reasons include the length of the phone calls, participant fatigue, and the structure of the qualitative questions. Adjustments such as shorter interviews, simplified questions, and separating quantitative and qualitative stages may improve adherence in future studies^(41)^.

The choice of the STAI-S6 was based on its brevity, validation, and feasibility for use in telephone-based interventions, which are essential features in PHC contexts. Nevertheless, it is important to recognize that this instrument may not capture the full complexity of anxiety symptoms, and combining it with other measures could enrich future analyses. Moreover, equity issues should be addressed: individuals with hearing impairments, cognitive difficulties, or without access to a telephone were excluded, which may limit the generalizability of the findings. Future adaptations could explore complementary strategies such as video calls, sign language resources, or in-person follow-up to ensure broader inclusivity^(42)^.

### Limitations of the Study

This study has some limitations. First, the absence of a control group limits the ability to establish causal inferences, but it is important to state that the objective of the feasibility study is to evaluate the feasibility of the intervention, not to evaluate its effectiveness. Second, there was considerable attrition during follow-up, particularly in the qualitative stage, which reduced the depth of the acceptability analysis. Third, the use of the STAI-S6, although validated and practical, may not capture the multidimensional nature of anxiety symptoms. Finally, the exclusion of groups such as individuals with hearing impairments, cognitive difficulties, or without access to a telephone highlights equity challenges that need to be addressed in future adaptations. These limitations are consistent with those observed in other digital health feasibility trials conducted in Brazil^(37)^.

### Advancements in the Field of Nursing

RIA has demonstrated potential to reduce symptoms of anxiety and is particularly useful in PHC, where the demand for mental health care is high and resources are often scarce. Through the telephone-based intervention, RIA allows health professionals, especially nurses, to monitor patients’ progress continuously and efficiently. This optimizes care time, facilitates clinical management, and increases access to personalized care. By integrating technologies such as RIA into nurses’ daily routines, it becomes possible to offer support that is more agile, scalable, and focused on patients’ needs.

## CONCLUSION

This feasibility study demonstrated that a phone-based intervention for anxiety management, grounded in Peplau’s Theory of Interpersonal Relations, is acceptable, practical, and has the potential to be integrated into the routine of PHC services. The high adherence, positive user feedback, and preliminary effectiveness in reducing anxiety symptoms support its viability as a low-cost and scalable strategy.

The findings also highlight the importance of professional training, which ensured fidelity to the protocol and points to the possibility of incorporating this practice into continuing education programs for nurses and into the training of undergraduate nursing students. Furthermore, the integration of RIA into PHC may be enhanced by aligning it with existing mental health screening protocols and digital health strategies within SUS.

Despite its limitations, this intervention represents a promising approach to expand access to mental health care in PHC, particularly in contexts of social vulnerability and limited access to specialized services. Future studies, particularly randomized controlled trials, will be essential to confirm its effectiveness and inform the development of public health policies. In addition, strategies to reduce participant attrition, ensure equity of access, and strengthen links with national digital health initiatives will be fundamental to consolidate its implementation and impact.

## Data Availability

The entire dataset supporting the results of this study was published in the article itself.
