# Supplementary material for: Phone-based intervention for anxiety management in primary health care users: A feasibility study
Source: Rev Esc Enferm USP. 2026 Mar 2;60:e20250255. doi: 10.1590/1980-220X-REEUSP-2025-0255en (PMC13004578; doi:10.1590/1980-220X-REEUSP-2025-0255en)
Supplement: Translation

# Translation: Intervención telefónica para el manejo de la ansiedad en usuarios de la atención de salud primaria: Un estudio de viabilidade^*^

**DOI:** 10.1590/1980-220X-REEUSP-2025-0255es

**Authors:** Caroline Figueira Pereira, Maria Paula Bortoleti de Araújo, Divane de Vargas, Maria Luiza Prado Uelze, Geoffrey Maina

**Affiliations:** 1 Universidade de São Paulo, Escola de Enfermagem, São Paulo, SP, Brasil.; 2 University of Saskatchewan, College of Nursing, Prince Albert, Saskatchewan, Canada.

**Keywords:** Estudios de Factibilidad, Ansiedad, Atención Primaria de Salud, Salud Mental

## Abstract

**Objetivo:**

Evaluar la viabilidad de una intervención telefónica basada en la teoría de las relaciones interpersonales de Peplau para el manejo de la ansiedad entre los usuarios de la atención de salud.

**Método:**

Se llevó a cabo un estudio cuasi-experimental, de un solo grupo, antes y después, en cuatro unidades de atención de salud de la ciudad de São Paulo (Brasil), entre enero y noviembre de 2021. Se evaluó la viabilidad en cuatro ámbitos: demanda, aceptabilidad, implementación y practicidad. Se analizó la teoría de las relaciones interpersonales para el manejo de la ansiedad mediante una intervención telefónica y su eficacia preliminar utilizando un modelo lineal de efectos mixtos.

**Resultados:**

Se reclutó a un total de 1327 adultos, de los cuales 1171 presentaban niveles de ansiedad de moderados a graves, identificados mediante el Inventario de Ansiedad Estado-Rasgo (the State-Trait Anxiety Inventory) - Forma Corta de Seis Ítems. Se observó una alta demanda (88,2 %) y una buena aceptabilidad, y el 69,8 % de los participantes completaron el seguimiento. Se observó una reducción significativa en las puntuaciones medias de ansiedad inmediatamente después de la intervención (p < 0,001).

**Conclusión:**

La intervención telefónica demostró su viabilidad en términos de demanda, aceptabilidad, implementación y practicidad, posicionándose como una estrategia prometedora para ampliar el acceso a la atención de salud mental en la atención primaria.

## INTRODUCCIÓN

La Organización Mundial de la Salud (OMS) define la salud mental como un estado de bienestar en el que las personas pueden desarrollar sus capacidades, hacer frente a las tensiones normales de la vida, trabajar de manera productiva y contribuir a sus comunidades. Los trastornos mentales pueden afectar profundamente a las relaciones sociales, el funcionamiento ocupacional y la salud física^(1)^. Según la OMS, aproximadamente el 20 % de las personas de hasta 25 años experimentan problemas de salud mental, casi el doble de la prevalencia observada en la población general^(2)^. Es alarmante que más del 85 % de estas personas, especialmente en los países de ingresos bajos y medios, no reciban una atención adecuada debido a las barreras que impiden el acceso a los servicios de salud mental^(3)^. Entre los trastornos mentales, la depresión y la ansiedad son los más prevalentes en todo el mundo^(4)^. Brasil ocupa el cuarto lugar entre los países con las tasas más altas de prevalencia de trastornos de ansiedad^(4)^. A pesar de esta alta prevalencia, solo el 23 % de las personas diagnosticadas con trastornos de ansiedad en Brasil tienen acceso a una atención médica adecuada, lo que compromete directamente su calidad de vida^(4)^.

La recolección de datos para este estudio se llevó a cabo en 2021, el segundo año de la pandemia de COVID-19, un contexto en el que se intensificaron los síntomas de ansiedad en la población brasileña y se sobrecargaron los servicios de salud mental. Los estudios indican que, durante la pandemia, la prevalencia de los síntomas de ansiedad en Brasil alcanzó niveles superiores al 40 %, especialmente entre las mujeres y los adultos jóvenes, grupos que mostraron una mayor vulnerabilidad al malestar psicológico^(5,6)^. Este escenario puso de relieve la necesidad de estrategias rápidas, accesibles y escalables para la atención de la salud mental, en particular en la atención primaria de salud (APS), la principal puerta de acceso al Sistema Único de Salud (SUS) brasileño.

Al mismo tiempo, se produjo un esfuerzo político e institucional hacia la transformación digital del SUS, con la implementación y expansión de programas como Telesalud, e-SUS y la Estrategia de Salud Digital para Brasil 2020–2028^(7,8)^. Estas iniciativas buscaban ampliar el acceso, reducir las barreras geográficas y fortalecer la resolución de la APS, haciendo hincapié en el uso de intervenciones remotas como alternativas a la atención presencial. En este contexto, investigar la viabilidad de intervenciones como la Relación Interpersonal en Ansiedad (RIA) mediante intervención telefónica cobra relevancia tanto como respuesta de emergencia a la crisis sanitaria como contribución a la consolidación de modelos innovadores de atención de salud mental en el SUS^(9,10)^.

La telesalud se ha convertido en una herramienta estratégica para fortalecer las redes de atención de salud en Brasil, mejorando los resultados de salud pública al reducir las barreras geográficas y facilitar la prestación de intervenciones digitales eficaces^(11)^. Los enfoques digitales para la detección y el manejo de la ansiedad han mostrado resultados prometedores en la atención de la salud mental. En el contexto actual, en el que la demanda de atención de salud mental ha aumentado significativamente, especialmente debido al incremento de los síntomas de ansiedad, es esencial explorar el potencial de las herramientas digitales para manejar estos síntomas^(12)^.

La RIA es una intervención estructurada y centrada en el cliente desarrollada por Pereira^(13)^, diseñada para apoyar el manejo de los síntomas de ansiedad a través de una intervención telefónica^(13)^. Este enfoque es particularmente adecuado para las personas que reciben atención en entornos de atención primaria de salud, especialmente en contextos de bajos ingresos donde el acceso a servicios especializados de salud mental es limitado^(13)^. La intervención RIA se basa en la teoría de las relaciones interpersonales de Peplau, que hace hincapié en la relación terapéutica como elemento central en el manejo del malestar psicológico. Adaptada originalmente del modelo teórico de la teoría interpersonal para el manejo de la ansiedad en personas con trastornos por consumo de sustancias^(13,14)^, la RIA se reestructuró para abordar a la población más amplia que experimenta ansiedad.

Como intervención compleja e interactiva, la RIA tiene como objetivo ayudar a las personas a conceptualizar su experiencia de ansiedad y a desarrollar estrategias de afrontamiento eficaces. Comprende cinco componentes esenciales: (1) promover la conciencia de la ansiedad, (2) facilitar la denominación o articulación de los sentimientos de ansiedad, (3) identificar los desencadenantes personales de la ansiedad, (4) reconocer los comportamientos de alivio existentes y (5) ofrecer un menú de alternativas de afrontamiento más saludables. Estos elementos están diseñados para mejorar la alfabetización emocional y empoderar a las personas para que respondan de manera más adaptativa a las situaciones que provocan ansiedad^(13,14)^. La Cuadro 1 ofrece un resumen de los elementos clave de la intervención RIA.

**Cuadro 1:** Elementos clave de la Relación Interpersonal en la Ansiedad (RIA) – São Paulo, SP, Brasil, 2025.^(13,15)^

Componentes	Contenido
Componente 1: Conciencia de la ansiedad	Proporcionar información sobre los niveles de ansiedad utilizando el Inventario de Ansiedad Estado-Rasgo – Formulario Corto de Seis Ítems (STAI-S6) Ofrecer psicoeducación sobre los síntomas de la ansiedad
Componente 2: Nombrar la ansiedad	Ayudar al participante a reconocer y aceptar su nivel de ansiedad moderado o grave.
Componente 3: Identificación de los desencadenantes de la ansiedad	Facilite la identificación de los desencadenantes personales asociados con la ansiedad. Explorar los determinantes de la ansiedad
Componente 4: Identificación de comportamientos de alivio	Ayudar al participante a reconocer los comportamientos actuales que utiliza para aliviar la ansiedad Promover el análisis de la relación entre los desencadenantes y las conductas de alivio
Componente 5: Menú de comportamientos saludables de alivio	Presentar un menú de estrategias saludables y basadas en la evidencia para el manejo de la ansiedad. Desarrollo de habilidades para manejar la ansiedad. Desarrollo de la autoeficacia para manejar la ansiedad.

Durante la sesión telefónica, se aplica inicialmente el Inventario de Ansiedad Estado-Rasgo – 6 ítems (State-Trait Anxiety Inventory – 6 items -STAI-S6) para evaluar el estado de ansiedad actual del participante. Posteriormente, se lleva a cabo la intervención RIA de forma secuencial, siguiendo sus cinco pasos estructurados. Tras la intervención, se vuelve a aplicar el STAI-S6 para identificar posibles cambios en los niveles de ansiedad, lo que permite evaluar de forma inmediata la eficacia de la intervención. Todo el proceso, incluyendo tanto la escala como la intervención, dura aproximadamente 5,7 minutos. Además, después de siete días, se vuelve a aplicar la RIA junto con el STAI-S6 para verificar su eficacia a medio plazo, como se ilustra en la Figura 1 a continuación. Este formato breve y estructurado mejora la viabilidad de la RIA en contextos de atención primaria.

**Figura 1:** Diagrama de flujo que ilustra la aplicación paso a paso de la intervención telefónica (RIA): evaluación inicial con STAI-S6 (T0), protocolo RIA estructurado (cinco pasos), reaplicación inmediata de STAI-S6 (T1) y llamada de seguimiento con reaplicación de STAI-S6 después de 7 días (T2) – SP, Brasil, 2025.

A pesar de los diversos retos relacionados con el acceso a la salud, la APS en Brasil ha empleado diversas estrategias para llegar a las poblaciones vulnerables. Entre ellas se incluyen el fomento de vínculos estrechos entre los pacientes y los equipos de atención médica, la oferta de atención integral a todos los grupos de edad, la realización de actividades comunitarias de promoción y prevención de la salud y la realización de visitas domiciliarias^(16)^. Sin embargo, la falta de herramientas adecuadas, el apoyo organizativo limitado y la formación insuficiente de los profesionales de la salud suelen obstaculizar la prestación de una atención de salud mental cualificada. Estas barreras son especialmente evidentes en el tratamiento de la ansiedad, donde es esencial una comunicación eficaz y empática^(17)^.

En este contexto, el presente estudio tuvo como objetivo evaluar la viabilidad y la eficacia preliminar de la RIA mediante una intervención telefónica para el tratamiento de los síntomas de ansiedad en adultos usuarios de los servicios de APS. Se espera que los resultados sirvan de base para el diseño y la estimación de parámetros de un futuro ensayo controlado aleatorio (ECA) a gran escala.

## MÉTODO

### Diseño del Estudio

Se trata de un estudio cuasi-experimental, de un solo grupo, antes y después, que evaluó la viabilidad^(18)^ de un protocolo de intervención remota por teléfono para el manejo de la ansiedad en adultos de cuatro unidades de APS en São Paulo, Brasil.

Se reclutó a un total de 1327 personas durante la programación de citas en las unidades. STAI-S6 Tras obtener el consentimiento informado, se recolectaron datos sociodemográficos y el equipo de investigación, formado por enfermeras y estudiantes de enfermería, administró el STAI-S6. El equipo recibió formación mediante la lectura de un manual de intervención y sesiones de juego de roles con el líder del equipo.

Los participantes con niveles de ansiedad de moderados a graves recibieron la intervención basada en la RIA. Teniendo en cuenta su efecto a medio plazo^(19)^, una semana después se llevó a cabo una intervención telefónica para volver a administrar el STAI-S6 y evaluar la eficacia de la intervención.

El marco teórico para el desarrollo de la RIA fue la teoría explicativa de alcance medio^(7)^. La intervención se describió de acuerdo con la lista de verificación TIDieR (Template for Intervention Description and Replication - Plantilla para la Descripción y Replicación de Intervenciones), que amplía las directrices CONSORT y SPIRIT al proporcionar normas claras para informar sobre las intervenciones en cuanto a la justificación, los materiales, los procedimientos, los proveedores y las estrategias de adherencia, lo que mejora la transparencia y la reproducibilidad^(20)^. El manual de la intervención se elaboró para guiar a los interventores a través de un proceso paso a paso y está disponible como material complementario.

### Escenario y Población

En Brasil, la APS adopta un enfoque dinámico y centrado en la comunidad, que se caracteriza por un profundo conocimiento del territorio, unas sólidas relaciones entre pacientes y proveedores, una atención integral y actividades proactivas de promoción de la salud y prevención de enfermedades. Las visitas domiciliarias son esenciales para monitorear las necesidades de los pacientes. Un estudio destaca que esta estructura ha sido crucial en situaciones de emergencia en el país^(16)^. Por esta razón, la población objetivo del estudio está formada por adultos de entre 18 y 90 años que son monitoreados (monitoreados) por las unidades de APS de la ciudad de São Paulo.

Las unidades de APS ubicadas en los barrios de Sé, Cambuci, República y Santa Cecília, todos ellos situados en la ciudad de São Paulo, en el estado de São Paulo (Brasil), fueron seleccionadas para este estudio debido a su ubicación en zonas de bajos ingresos caracterizadas por la vulnerabilidad social y los complejos retos que plantea la atención a los pacientes.

### Criterios de Inclusión y Exclusión

Los criterios de inclusión fueron: (a) ser mayor de 18 años, (b) haber acudido a una consulta en la unidad de APS, (c) comprender el portugués lo suficiente como para entender las preguntas del entrevistador, (d) presentar una puntuación igual o superior a 11 en la escala STAI-S6, y (e) estar disponible para el seguimiento. Los criterios de exclusión fueron: (a) estar en tratamiento por problemas relacionados con el consumo de alcohol y/u otros trastornos mentales, (b) pacientes que mostraran signos evidentes de confusión mental o intoxicación por sustancias psicoactivas durante la llamada telefónica, (c) personas con discapacidad auditiva, y (d) pacientes que se negaran a recibir la intervención y/o el seguimiento.

### Muestra y Procedimientos de Recolección

Reclutamos a 1327 usuarios de la APS entre enero de 2021 y noviembre de 2021 a través del calendario de consultas y asistencia de las unidades de APS participantes. El objetivo era obtener una muestra de 75 participantes, ya que este número es aceptable para un estudio de viabilidad ^(14,21)^.

La muestra se seleccionó por conveniencia, y los intervinientes capacitados se pusieron en contacto con los posibles participantes por teléfono para explicarles el estudio. Toda la recolección de datos se realizó a través de llamadas telefónicas de 20 a 30 minutos, durante las cuales se recolectaron datos sociodemográficos, clínicos y de comportamiento, se administró el STAI-S6 y se llevó a cabo la intervención telefónica cuando procedía. Los datos se introdujeron directamente en la aplicación de investigación y en la plataforma de captura electrónica de datos (REDCap), una plataforma web segura para la gestión de datos de investigación.

La información para recolectar incluía el sexo, la raza, el estado civil, el empleo, la educación, las comorbilidades (diabetes, hipertensión, COVID-19) y la actividad física. Se utilizó el STAI-S6, una escala validada de seis ítems (α de Cronbach: 0,93 para los hombres y 0,87 para las mujeres)^(22)^, para detectar la ansiedad, con puntuaciones que oscilaban entre 6 y 10 (leve), 11 y 15 (moderada) y 16 y 24 (grave).

Los participantes elegibles recibieron la RIA mediante una intervención telefónica (T0), seguida inmediatamente de una segunda aplicación del STAI-S6 (T1) para evaluar el impacto a corto plazo (intervalo medio: 5,7 minutos). Se realizó una llamada de seguimiento después de siete días (T2) para reevaluar y evaluar los efectos sostenidos.

### Análisis y Tratamiento de Datos

Los datos se recolectaron y gestionaron en REDCap y se analizaron en el programa estadístico R versión 4.0.2^(23)^. El análisis descriptivo se realizó mediante el análisis de frecuencias absolutas y relativas. Para evaluar la eficacia preliminar de la RIA, se utilizó el modelo lineal de efectos mixtos. El nivel de significación adoptado fue del 5 %.

### Aspectos Éticos

Este estudio fue aprobado por el Comité de Ética de la Facultad de Enfermería de la Universidad de São Paulo (24461219.9.0000.5392/2019) y por la Secretaría Municipal de Salud de São Paulo (4.342.492). Además, se obtuvo el consentimiento informado verbal de todos los participantes, que fue registrado. El procedimiento de consentimiento informado verbal fue aprobado por el Comité de Ética de la Facultad de Enfermería de la Universidad de São Paulo. La participación fue voluntaria y se informó a los participantes de que podían retirarse del estudio en cualquier momento. Todos los métodos utilizados en este estudio siguieron las directrices y normativas del Comité de Ética.

## RESULTADOS

### Participantes

Se evaluó la elegibilidad de un total de 1327 adultos. De ellos, 144 personas (10,85 %) se negaron a participar, alegando en la mayoría de los casos falta de interés en el tema del estudio. La muestra final estuvo compuesta por 1183 participantes, de los cuales 710 eran mujeres (60,01 %), 472 eran hombres (39,89 %) y un participante (0,01 %) optó por no revelar su género.

La mayoría de los participantes eran solteros (n = 443; 38,06 %) y se identificaban como blancos (n = 485; 41,00 %). La edad media de la muestra era de 48,97 años. En cuanto a la educación, una parte importante había completado la preparatoria (n = 425; 37,09 %) y 411 personas (35,31 %) declararon estar empleadas en el momento del estudio.

### Evaluación de la Viabilidad

Se evaluaron medidas sólidas de viabilidad, como la demanda, la aceptabilidad, la implementación y la practicidad^(21,24)^. La Cuadro 2 nos informa de los componentes que se utilizaron para evaluar la viabilidad de la intervención RIA.

**Cuadro 2:** Evaluación de los componentes de la viabilidad del programa RIA – São Paulo, SP, Brasil, 2025.

Componentes	Indicadores
Demanda	Disposición a participar: (%) de participantes que aceptaron y (%) que no aceptaron participar en el estudio Selección: (%) de participantes que no son elegibles según los criterios de inclusión
Aceptabilidad	Tasas de seguimiento: (%) de participantes que completan el seguimiento. Tasas de datos faltantes: (%) de datos faltantes. Desgaste: (%) de participantes que participan en la intervención a lo largo de los periodos de tiempo (T0, T1 y T2)
Implementación	Diseño: evaluación de las respuestas a la pregunta: «¿Hay algo que cambiaría de la intervención que recibió por teléfono?».
Practicidad	Capacidad de los participantes para implementar estrategias de RIA para manejar la ansiedad.

El primer criterio de viabilidad, la demanda, se evaluó calculando la proporción de personas que aceptaron participar tras el proceso de selección inicial. Se seleccionó a un total de 1327 adultos según los criterios de elegibilidad del estudio. De ellos, 144 personas (10,85 %) rechazaron participar, y la mayoría (n = 46; 70,77 %) alegó falta de interés en el tema. Como resultado, 1183 personas aceptaron inicialmente participar. De ellas, 1171 cumplían todos los criterios de elegibilidad, que incluían el consentimiento para participar y presentar síntomas de ansiedad de moderados a graves según la escala STAI-S6. Esto dio lugar a una tasa de participación final del 88.2 %, lo que indica una alta demanda de la intervención.

El componente 2 (aceptabilidad) se evaluó mediante las tasas de seguimiento, las tasas de ausencias y el desgaste. Al realizar un análisis descriptivo del STAI-S6, es posible verificar la distribución de los sujetos que respondieron a las llamadas. En la fase de selección, se contactó con 1327 (100 %) personas, de las cuales 1183 (89,14 %) aceptaron participar en la investigación. De ellas, 1171 (88,22 %) presentaban ansiedad moderada o grave y, por lo tanto, aceptaron la intervención RIA (T0). Durante la intervención, de los 1171 participantes que aceptaron participar, 606 personas (51.75 %) comenzaron a responder al STAI-S6 después de la intervención (T1), pero solo 468 (77.22 %) respondieron completamente al STAI-S6 después de la intervención. De estas 468 personas, el 72 % presentaba ansiedad moderada y el 28 % presentaba ansiedad grave. Finalmente, de los participantes que completaron las escalas, 327 (69,87 %) completaron el seguimiento, es decir, respondieron a la segunda llamada y respondieron a la escala STAI-S6 siete días después de la aplicación de la intervención.

La implementación (componente 3) se evaluó preguntando a los participantes al final de la llamada de seguimiento si sugerirían algún cambio en la intervención. Cabe señalar que de los 1171 participantes, solo 92 respondieron a esta pregunta. De ellos, 72 (78,26 %) informaron que no cambiarían la intervención recibida, lo que demuestra una buena aceptación de la RIA. A pesar de ello, se abordaron algunas sugerencias durante la intervención telefónica, como que la intervención fuera presencial (n = 4, 4,35 %) y que se trataran cuestiones más subjetivas (n = 3, 3,26 %). Cabe mencionar que la gran mayoría de los participantes (n = 1091, 92,22 %) no respondió a esta pregunta, lo que debe tenerse en cuenta en la evaluación de la implementación y, sobre todo, para mejorar el estudio.

En cuanto al componente 4 de viabilidad (practicidad), la evaluación de este componente incluyó la capacidad de los participantes para implementar las estrategias de manejo de la ansiedad sugeridas durante la implementación de la RIA, como el ejercicio físico, las técnicas de respiración, la buena nutrición, escuchar música, las manualidades y la conversación con amigos y familiares, así como evaluar si eran útiles. Los resultados mostraron que, de las 92 personas que respondieron a esta pregunta, el 70.65 % (n = 65) de las personas informaron que pudieron implementar las técnicas en su vida diaria y que les ayudaron a manejar la ansiedad.

### Eficacia Preliminar

Los resultados sugieren un efecto positivo de la RIA en la reducción de los síntomas de ansiedad entre los participantes, se observaron diferencias estadísticamente significativas entre los momentos de aplicación de la escala STAI-S6, con una disminución de 0.45 puntos en promedio en T1, sin embargo, no se observó ningún cambio en las puntuaciones en T2 (Tabla 1). Estos resultados son coherentes con los presentados en la Tabla 2, que indican que la diferencia entre los momentos del estudio fue significativamente mayor entre T0 y T1, lo que no se observa en la comparación con T2. Sin embargo, al tratarse de un estudio de viabilidad, no se utilizó un grupo de control para verificar la eficacia de la RIA. Estas cuestiones se aclararán en el futuro en un ensayo clínico aleatorizado.

**Tabla 1:** Resultado del modelo lineal de efectos mixtos para la comparación del STAI-S6^*^ en la línea de base, T1 y T2 con respecto a la RIA para la ansiedad – São Paulo, SP, Brasil, 2025.

Tiempo	N	Media	Valor p ^†^
Línea de base (T0)	117 1	12,47	
STAI-S6 posterior (T1)	468	11,23	<0,001
Seguimiento (T2)	327	10,86	

^*^STAI-S6 = Escala de Inventario de Ansiedad Rasgo -S6;

^††^ Modelo lineal de efectos mixtos

**Tabla 2:** Comparación secuencial Dos X Dos de las tres medidas del STAI-S6^*^ en la línea de base, T1 y T2 con respecto al RIA para la ansiedad – São Paulo, SP, Brasil, 2025

Tiempo/Intervención	Coeficiente	SE	IC inf.	IC sup.	Valor p
STAI-S6^*^ inicial (T0) frente a STAI-S6 posterior (T1)	–0,453	0,146	–0,739	–0,166	0,002
STAI-S6 post^*^ (T1) frente a STAI-S6^*^ (T2)	–0,005	0,176	–0,350	0,341	0,979

## DISCUSIÓN

Los cambios drásticos en la vida cotidiana y el aumento de las situaciones estresantes a lo largo de los años han tenido graves consecuencias para la salud mental de la población, como el aumento de los trastornos de ansiedad, tal y como han documentado diversos autores^(5,6,7)^. En vista de esta situación, cada vez es más necesario monitorear de forma estrecha la salud mental de la población, dado el alto riesgo de malestar psicológico y el hecho de que muchas personas pueden tener dificultades para hacer frente a estas adversidades^(7)^.

Los resultados de este estudio reflejan este contexto más amplio, indicando un número representativo de personas con síntomas de ansiedad de moderados a graves. Una encuesta nacional reveló que el 40 % de los adultos informaban de sentimientos frecuentes de tristeza y, al analizar específicamente los síntomas de ansiedad, esta cifra se acerca al 50 %^(8)^. Además, la tristeza y la ansiedad son especialmente frecuentes entre las mujeres y los adultos jóvenes^(25)^, un patrón que también se observa en este estudio. Estas observaciones refuerzan la urgencia de desarrollar estrategias específicas para abordar la ansiedad, especialmente en entornos de atención médica de alto alcance, como la atención primaria.

En este sentido, el perfil sociodemográfico de los participantes en el estudio también revela determinantes estructurales y sociales que pueden influir no solo en la aparición y la gravedad de la ansiedad, sino también en la viabilidad de aplicar estrategias de control de la ansiedad en la vida cotidiana. En nuestra muestra predominaban las mujeres (60,01 %), las personas que se identificaban como blancas (41,00 %) y los participantes solteros (37,44 %). Una parte considerable había completado la preparatoria (37,09 %) y una proporción ligeramente mayor declaró estar empleada (35,31 %) frente a los desempleados (29,98 %).

Estos indicadores ponen de relieve importantes determinantes sociales de la salud mental. Las pruebas sugieren que las mujeres se ven más afectadas por los trastornos de ansiedad debido a múltiples factores, como las variaciones hormonales y una mayor exposición a las responsabilidades domésticas y de cuidado^(25)^. Del mismo modo, el desempleo se asocia con el malestar psicológico, a menudo vinculado a la inseguridad financiera y a la ausencia de una rutina estructurada^(26)^. El nivel educativo también desempeña un papel determinante, ya que influye en el acceso a la información sanitaria, la comprensión de las recomendaciones de autocuidado y la capacidad de integrar nuevas prácticas en la vida cotidiana.

Cuando se analizan en relación con la viabilidad de la RIA, estos determinantes pueden facilitar o dificultar la adopción de las estrategias sugeridas, como la actividad física, la alimentación saludable o las técnicas de relajación. Por ejemplo, las personas con recursos económicos limitados pueden encontrar obstáculos para mantener una nutrición adecuada o acceder a espacios para hacer ejercicio, mientras que aquellas con mayores recursos educativos pueden tener más facilidad para comprender y aplicar técnicas de respiración u otras estrategias de afrontamiento. Por lo tanto, más allá de la motivación individual, el contexto social es un factor decisivo a la hora de determinar cómo se experimentan y se mantienen las intervenciones^(27,28)^.

Estos hallazgos subrayan la necesidad de estrategias de salud pública que sean sensibles a las vulnerabilidades sociodemográficas, garantizando que intervenciones como la RIA sean adaptables e inclusivas, especialmente en el ámbito de la APS^(27,28)^.

La APS desempeña un papel central como principal puerta de acceso al SUS de Brasil. Se encarga de recibir, monitorear y coordinar la atención de los pacientes a lo largo del tiempo y es capaz de atender hasta el 90 % de las demandas de salud de la población^(29)^. Por lo tanto, la implementación de estrategias sistemáticas para detectar los síntomas de ansiedad en las Unidades Básicas de Salud (UBS) puede promover la detección temprana y las intervenciones oportunas, previniendo la escalada de los síntomas y reduciendo la necesidad de derivaciones a servicios especializados^(30)^.

El fortalecimiento de la capacidad de los servicios de atención primaria para manejar trastornos mentales comunes como la ansiedad también puede desempeñar un papel decisivo en la prevención de la sobrecarga de los servicios especializados de salud mental. Las intervenciones psicosociales breves, como la psicoeducación, las estrategias de terapia cognitivo-conductual adaptadas a la APS y el uso de tecnologías digitales, han demostrado su eficacia en el manejo de casos de ansiedad leve a moderada^(31)^. Las pruebas obtenidas en Brasil refuerzan la viabilidad de las intervenciones psicosociales en entornos de APS, como la atención plena y la terapia cognitivo-conductual basada en Internet^(32,33)^. La integración de estos enfoques en el trabajo diario de los equipos de la Estrategia de Salud Familiar (ESF) amplía el acceso a la atención, promueve la autonomía de los usuarios y fortalece la capacidad de resolución de problemas de la red de atención primaria. Esta perspectiva está en consonancia con los principios de la Red de Atención Psicosocial (RAPS) y las directrices de las políticas públicas de salud mental de Brasil^(34)^.

La intervención estructurada para el manejo de la ansiedad (RIA) fue llevada a cabo por enfermeras y estudiantes de enfermería que recibieron capacitación específica para aplicar el protocolo. La teoría de Peplau destaca la importancia de la relación terapéutica como mediadora en el proceso de afrontamiento del malestar psicológico, lo que refuerza la idoneidad de este marco para la práctica de la salud mental en la APS^(35)^. La capacitación de los profesionales incluyó talleres a distancia, juegos de rol y supervisión continua, lo que garantizó el cumplimiento del protocolo y reforzó su viabilidad. Estrategias similares han demostrado su eficacia en intervenciones digitales de salud mental realizadas en América Latina^(36,37)^. Esta característica también sugiere que la intervención podría incorporarse a los programas de educación continua para enfermeras de APS, lo que contribuiría a la sostenibilidad de la propuesta.

Para que la RIA se incorpore de manera sostenible a las rutinas de la APS, especialmente dentro de la FHS, son necesarias algunas adaptaciones. Entre ellas se incluyen: (1) formación específica para las enfermeras a fin de garantizar la fidelidad en la aplicación del protocolo; (2) consideración de las limitaciones de tiempo y la sobrecarga de trabajo a las que se enfrentan los equipos de APS; (3) la integración con los protocolos de detección de salud mental existentes para evitar duplicaciones y optimizar los flujos de trabajo; y (4) el uso de tecnologías de salud digitales dentro del SUS, como los registros médicos electrónicos de la atención primaria (e-SUS) y los programas de telesalud (Telessaúde), que son herramientas estratégicas para la escalabilidad, el monitoreo continuo y la sostenibilidad de la intervención^(38,39)^.

Además, la descripción detallada de la intervención siguió las recomendaciones de la lista de verificación TIDieR, que proporciona una guía estructurada para garantizar la transparencia, la claridad y la reproducibilidad de las intervenciones en la atención de salud. Este rigor metodológico es fundamental para permitir la replicación de experiencias como la RIA en otros contextos de atención primaria, respetando al mismo tiempo las especificidades locales^(20)^.

La implementación de la intervención RIA propuesta en este estudio dentro de la APS demostró una eficacia inicial en la reducción de los síntomas de ansiedad, además de mostrar un gran potencial para llegar a la población. La alta adherencia y eficacia de las estrategias utilizadas durante las llamadas remotas refuerzan la viabilidad de la intervención. Estos hallazgos son coherentes con las pruebas que respaldan el uso de la intervención telefónica para identificar los síntomas de ansiedad y los factores agravantes, contribuyendo así a mejorar la calidad de vida de las personas. Sin embargo, es importante reconocer que, a pesar de las numerosas ventajas de la intervención telefónica, esta no sustituye al tratamiento presencial^(36)^.

En cuanto a los criterios de viabilidad evaluados en el estudio, la intervención satisfizo la demanda: 1183 personas aceptaron participar, de las cuales 1171 (88,2 %) cumplían los criterios de elegibilidad. Esto indica que una parte sustancial de la población atendida por la APS presentaba síntomas de ansiedad de moderados a graves. Según la literatura, incluso muestras más pequeñas (alrededor de 39 participantes) se consideran suficientes para los estudios de viabilidad y pueden servir de base para ajustar los perfiles clínicos y sociodemográficos de los participantes para futuros ensayos clínicos aleatorios^(40)^.

La aceptabilidad de la intervención RIA también fue notable, ya que el 69.87 % de los participantes completaron el seguimiento. Aunque hubo algunas pérdidas durante el seguimiento, las tasas se mantuvieron dentro de los límites aceptables para los ensayos clínicos^(41)^. El éxito de la implementación quedó demostrado por los altos niveles de satisfacción de los participantes con la intervención recibida, y su practicidad quedó demostrada por la facilidad con la que los participantes aplicaron las técnicas sugeridas por la RIA en su vida cotidiana. Cabe destacar que el 78.26 % de los participantes afirmaron que no cambiarían nada de la intervención, y el 70.65 % informaron que utilizaban habitualmente las técnicas propuestas para controlar la ansiedad. Sin embargo, es importante destacar que 1079 personas no respondieron a las preguntas subjetivas al final de la consulta, lo que afectó a la evaluación completa de la implementación y la viabilidad. Esta laguna puede deberse al cansancio de los participantes, ya que las preguntas se insertaron al final de la consulta y siguieron a la aplicación de varias escalas, una dificultad que se señala con frecuencia en los estudios de viabilidad^(41)^.

A la luz de estos retos, se recomienda adoptar un enfoque más pragmático para orientar las intervenciones complejas^(41)^, así como reorganizar el calendario y el formato de las aplicaciones de las escalas para reducir la fatiga de los participantes y mejorar las tasas de respuesta. Además, es esencial garantizar la seguridad de los datos, incluida su integridad, exactitud, confidencialidad, privacidad y secreto profesional, especialmente en el contexto de la telesalud, y debe tratarse como un aspecto central en la implementación de estrategias de atención de salud mental a distancia^(41)^.

El objetivo de un estudio de viabilidad es evaluar si la intervención funciona en un escenario real, lo que afecta a la inferencia causal, lo que dificulta determinar si los resultados observados se debieron exclusivamente a la intervención. Por estas razones, la eficacia preliminar debe analizarse con cautela. Serán necesarios futuros ensayos controlados aleatorios para confirmar su eficacia. Además, el desgaste de los participantes durante el seguimiento y la baja tasa de respuesta a las preguntas subjetivas limitaron la evaluación de la aceptabilidad y la practicidad. Entre las posibles razones se encuentran la duración de las llamadas telefónicas, el cansancio de los participantes y la estructura de las preguntas cualitativas. Ajustes como entrevistas más breves, preguntas simplificadas y la separación de las etapas cuantitativas y cualitativas podrían mejorar la adherencia en futuros estudios^(41)^.

La elección del STAI-S6 se basó en su brevedad, validación y viabilidad para su uso en intervenciones telefónicas, características esenciales en el contexto de la atención primaria. No obstante, es importante reconocer que este instrumento puede no captar toda la complejidad de los síntomas de ansiedad, y combinarlo con otras medidas podría enriquecer los análisis futuros. Además, deben abordarse las cuestiones de equidad: se excluyó a las personas con discapacidad auditiva, dificultades cognitivas o sin acceso a un teléfono, lo que puede limitar la generalización de los resultados. Las adaptaciones futuras podrían explorar estrategias complementarias, como videollamadas, recursos de lenguaje de señas o seguimiento presencial, para garantizar una mayor inclusividad^(42)^.

### Limitaciones del Estudio

Este estudio tiene algunas limitaciones. En primer lugar, la ausencia de un grupo de control limita la capacidad de establecer inferencias causales, pero es importante señalar que el objetivo del estudio de viabilidad es evaluar la viabilidad de la intervención, no su eficacia. En segundo lugar, se produjo un desgaste considerable durante el seguimiento, especialmente en la fase cualitativa, lo que redujo la profundidad del análisis de aceptabilidad. En tercer lugar, el uso del STAI-S6, aunque validado y práctico, puede no captar la naturaleza multidimensional de los síntomas de ansiedad. Por último, la exclusión de grupos como las personas con discapacidad auditiva, dificultades cognitivas o sin acceso a un teléfono pone de relieve los retos de equidad que deben abordarse en futuras adaptaciones. Estas limitaciones son coherentes con las observadas en otros ensayos de viabilidad de salud digital realizados en Brasil^(37)^.

### Avances en el Campo de la Enfermería

La RIA ha demostrado su potencial para reducir los síntomas de ansiedad y es particularmente útil en la atención primaria de salud, donde la demanda de atención de salud mental es alta y los recursos suelen ser escasos. A través de la intervención telefónica, la RIA permite a los profesionales de la salud, especialmente a las enfermeras, monitorear el progreso de los pacientes de manera continua y eficiente. Esto optimiza el tiempo de atención, facilita el manejo clínico y aumenta el acceso a la atención personalizada. Al integrar tecnologías como la RIA en las rutinas diarias de las enfermeras, es posible ofrecer un apoyo más ágil, escalable y centrado en las necesidades de los pacientes.

## CONCLUSIÓN

Este estudio de viabilidad demostró que una intervención telefónica para el manejo de la ansiedad, basada en la teoría de las relaciones interpersonales de Peplau, es aceptable, práctica y tiene el potencial de integrarse en la rutina de los servicios de atención primaria de salud. La alta adherencia, los comentarios positivos de los usuarios y la eficacia preliminar en la reducción de los síntomas de ansiedad respaldan su viabilidad como estrategia de bajo costo y escalable.

Los resultados también destacan la importancia de la formación profesional, que garantizó la fidelidad al protocolo y apunta a la posibilidad de incorporar esta práctica en los programas de educación continua para enfermeras y en la formación de los estudiantes de enfermería de pregrado. Además, la integración de la RIA en la APS puede mejorarse alineándola con los protocolos de detección de salud mental y las estrategias de salud digital existentes dentro del SUS.

A pesar de sus limitaciones, esta intervención representa un enfoque prometedor para ampliar el acceso a la atención de salud mental en la APS, especialmente en contextos de vulnerabilidad social y acceso limitado a servicios especializados. Los estudios futuros, en particular los ensayos controlados aleatorios, serán esenciales para confirmar su eficacia y orientar el desarrollo de políticas de salud pública. Además, las estrategias para reducir el desgaste de los participantes, garantizar la equidad en el acceso y fortalecer los vínculos con las iniciativas nacionales de salud digital serán fundamentales para consolidar su implementación e impacto.

## Data Availability

Todo el conjunto de datos que apoya los resultados de este estudio fue publicado en el propio artículo.
